# Effect of Moisture Content on the Processing and Mechanical Properties of a Biodegradable Polyester

**DOI:** 10.3390/polym13101616

**Published:** 2021-05-17

**Authors:** Vincenzo Titone, Antonio Correnti, Francesco Paolo La Mantia

**Affiliations:** 1 Department of Engineering, University of Palermo, Viale delle Scienze, 90128 Palermo, Italy; 2 INSTM Consortium for Materials Science and Technology, Via Giusti 9, 50125 Florence, Italy; 3 Joeplast S.p.A., Zona Industriale S.S. 189, 92025 Casteltermini, Italy; info.antocor@gmail.com

**Keywords:** biodegradable polymers, mechanical properties, rheology, processing, moisture content

## Abstract

This work is focused on the influence of moisture content on the processing and mechanical properties of a biodegradable polyester used for applications in injection molding. The pellets of the biodegradable polyester were exposed under different relative humidity conditions at a constant temperature before being compression molded. The compression-molded specimens were again placed under the above conditions before the mechanical testing. With all these samples, it is possible to determine the effect of moisture content on the processing and mechanical properties separately, as well as the combined effect of moisture content on the mechanical properties. The results obtained showed that the amount of absorbed water—both before processing and before mechanical testing—causes an increase in elongation at break and a slight reduction of the elastic modulus and tensile strength. These changes have been associated with possible hydrolytic degradation during the compression molding process and, in particular, with the plasticizing action of the moisture absorbed by the specimens.

## 1. Introduction

The increasing use of plastics in agriculture and the growing amount of land and sea debris have led to the design and development of new polymer materials more “friendly” to the environment [1,2,3].

Generally, these biodegradable polymers are more expensive than traditional ones but are more environmentally friendly and more suitable for several specific applications, such as food packaging, agricultural mulching films, etc. [4,5,6,7,8]. In particular, biodegradable and compostable polymers are very appealing when used for some applications in agriculture such as films for mulching and plastic pots because these products can be left in the ground where they are transformed into water, CO_2_, and biomass. This biomass is useful as a soil improver.

Most parts of the biodegradable polymer systems are made of polyesters; it is well known that the polyesters absorb humidity, and the presence of moisture gives rise to dangerous degradation phenomena. The degradation phenomena are mainly due to the hydrolysis of the macromolecular chains with a severe decrease of molecular weight [9,10,11]. In addition, the presence of moisture strongly modifies the mechanical properties [11,12,13].

Although the influence of temperature was widely studied in the literature [14,15,16,17,18,19], the influence of relative humidity on the processing and mechanical properties is still an open field of research.

Harris and Lee [11] investigated the hydrolytic degradation of PLA and a PLA/polycarbonate blend exposed at high temperature and humidity, finding a significant moisture absorption and hydrolysis, resulting in degradation of properties. Similarly, Muthuraj et al. [12] reported studies on the hydrolytic degradation of poly(butylene succinate), PBS, poly(butylene adipate-*co*-terephthalate), PBAT, and PBS/PBAT blend, finding that, as a result of chain scission because of the hydrolysis mechanism, mechanical performance was significantly affected after conditioning.

In our previous work [13], we demonstrated how the presence of moisture significantly affects the mechanical properties of biodegradable polyesters subjected to UV irradiation.

Of course, the application of fully amorphous biodegradable polymers is limited by the fact that the glass transition temperature (Tg) of the polymer is strongly influenced by the relative humidity, especially for hydrophilic polymers.

The aim of this study was to investigate the influence of moisture on the processing and mechanical properties of a biodegradable polyester used for injection molding. In particular, the effect of moisture has been investigated on both the pellets before processing and on the specimens before the mechanical testing. In particular, pellets of a biodegradable polyester were exposed at different relative humidity values and fixed temperatures before compression molding to investigate the effect of this treatment on the rheological behavior of this polymer. The compression-molded specimens were again treated under the same conditions before the mechanical testing in order to investigate the effect of the presence of different moisture contents both before and after processing on the mechanical properties.

The experimental results clearly indicate that the processing of humid samples led to a decrease in molecular weight and consequent decrease in viscosity. The mechanical properties are, of course, influenced by the presence of moisture absorbed both before and/or after the compression molding. In the conditions adopted in this work, the more important effect on the mechanical properties is the plasticizing effect of the moisture.

## 2. Materials and Methods

The material used in this study was a biodegradable polyester Mater-Bi TF01U supplied by Novamont (Novamont, Novara, Italy). It is a bioplastic based on an aliphatic polyester with a melting point of 72–75 °C and a glass transition temperature between −40 and −35 °C, used for injection molding.

The specimens for the rheological and mechanical characterization were prepared by compression molding in a Carver (Carver, Wabash, IN, USA) laboratory hydraulic press at the temperature of 180 °C under a mold pressure of 300 psi and for about 3 min. Before compression molding, the pellets were subjected to three different pretreatments in the environmental conditions reported in Table 1.

Condition 1 means, of course, a treatment in the dry state, while condition 3 is relative to treatment in an almost water-saturated condition. The temperature has been chosen to accelerate the sorption of the humidity.

Before testing the mechanical properties, the specimens were subjected to the same environmental conditions reported in Table 1.

**Figure 1:**
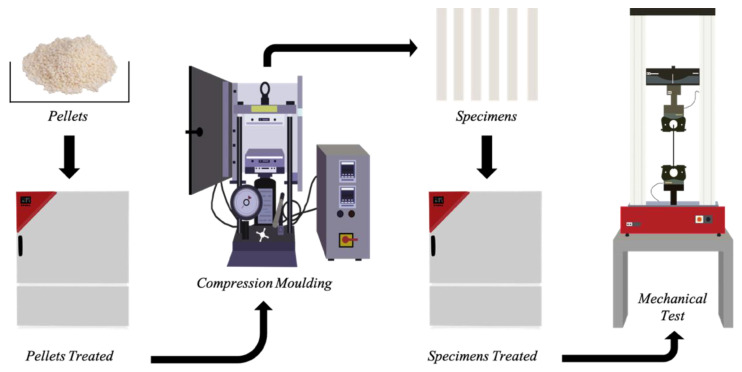
Scheme of the procedure adopted in this work.

Figure 1 depicts the processing undergone by the pellets and by the compression-molded specimens.

The conditioning of the pellets and of the compression-molded samples was carried out in a climate chamber KBF 115–Binder (Binder, Tuttlingen, Germany).

Samples conditioned in nine different conditions (Table 2) were then investigated.

The first subscript indicates under which conditions (Table 1**)** the pellets have been conditioned before processing and the second subscript indicates under which conditions the compression-molded specimens have been treated. For example, for the sample code MC_11_, the pellets have been treated under condition 1 before compression molding, and the compression-molded specimens were treated again under condition 1 before testing the mechanical properties, while, for the sample MC_23_, the pellets have been treated under condition 2 before compression molding and the compression-molded specimens were treated under condition 3 before testing the mechanical properties. With all these samples it is then possible to determine separately the effect of moisture content on the processing and mechanical properties, as well as the combined effect—processing and environmental exposure—on the mechanical properties of moisture content.

Moisture content was calculated according to ASTM D570-98 [20], using Equation (1).

(1) $$\mathrm{MC},\;\%=\frac{\left(W_{w}-W_{d}\right)}{W_{d}}\;*100$$

where *W_W_* and *W_d_* are the weight of the sample after and before moisture exposure, respectively.

The rheological characterization was performed on disk-shaped samples, using an ARES G2 (TA Instruments, New Castle, DE, USA). The experiments were carried out in parallel plates with a gap of about 1.5 mm and a diameter of 25 mm. The shear viscosity values of the samples were measured at 180 °C and in a frequency range from 0.1 and 100 rad/s.

Tensile properties tests were carried out using an Instron (Instron, High Wycombe, UK) universal testing machine mod. 3365 equipped with a 1 kN load cell and 30 mm gauge length extensimeter. The tensile strength specimens were rectangular sheets according to ASTM D638-14 [21] (length: 90 mm, width: 10 mm, thickness: ≃0.4 mm). The mechanical tests were carried out on the conditioned specimens immediately after the end of the conditioning in order to avoid any significant change in the value of the moisture.

Elastic modulus, E, tensile strength, TS, and elongation at break, EB, were measured, and the reported data were determined as an average of 12 samples. The elastic modulus was measured at a deformation speed of 1 mm/min. When the deformation achieved 10%, the crosshead speed was increased to 100 mm/min until final breaking.

## 3. Results and Discussion

In Table 3, the values of moisture content at equilibrium for all three conditioning treatments are reported. As expected, the amount of moisture increases with increasing the relative humidity.

**Figure 2:**
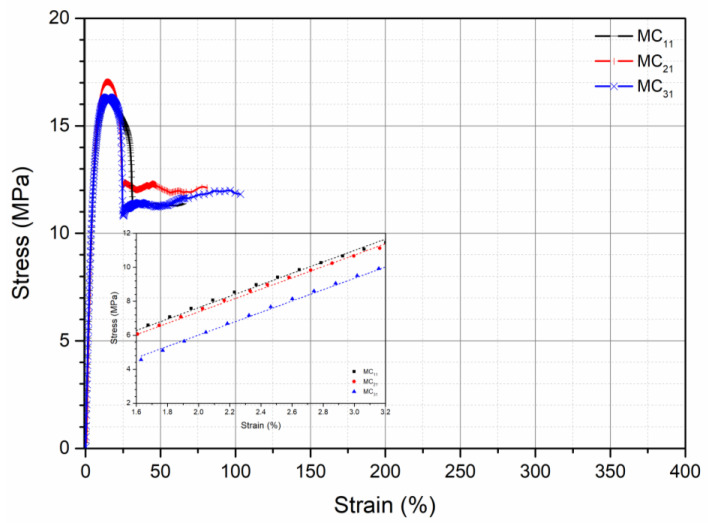
Stress–strain curve of samples coming from pellets conditioned under the three conditions and specimens for mechanical testing conditioned at RH = 0%.

**Figure 3:**
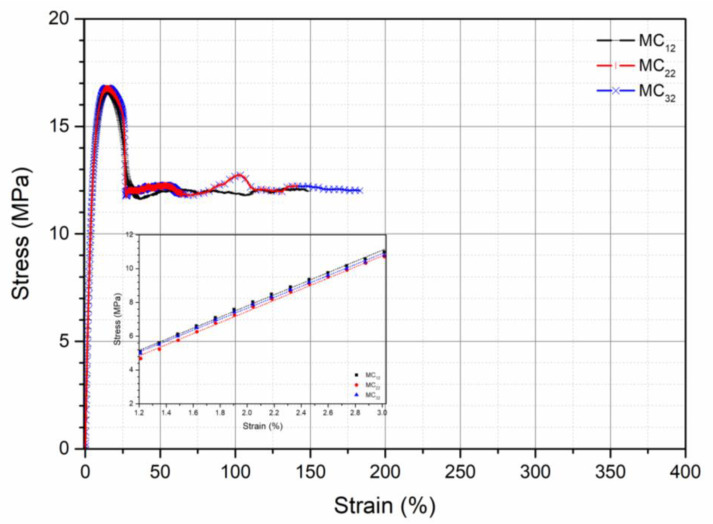
Stress–strain curve of samples coming from pellets conditioned under the three conditions and specimens for mechanical testing conditioned at RH = 50%.

**Figure 4:**
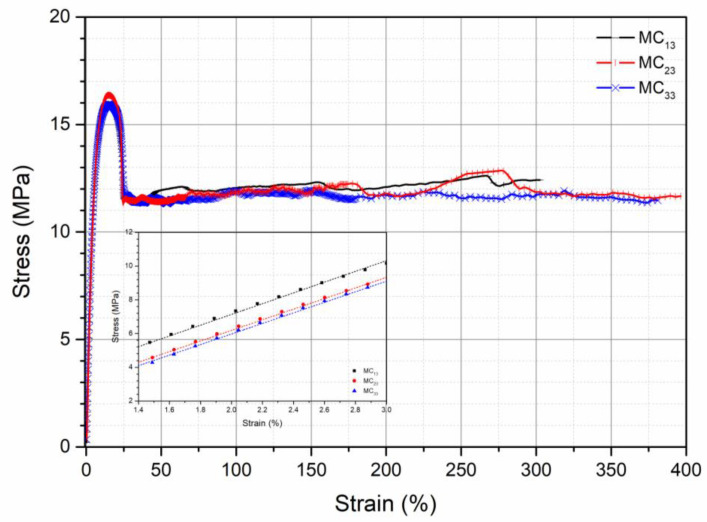
Stress–strain curve of samples coming from pellets conditioned under the three conditions and specimens for mechanical testing conditioned at RH = 90%.

Stress–strain curves obtained from the tensile tests of all the specimens are reported in Figure 2, Figure 3 and Figure 4.

All the curves show a ductile behavior, but the values of elastic modulus, E, tensile strength, TS, and elongation at break, EB are different according to the pretreatment, as evident in Table 4, in which the values of these mechanical properties for all the investigated samples are reported.

The elastic modulus is only slightly dependent on the pretreatment conditions both before the processing and before the mechanical testing. Tensile strength, on the contrary, depends significantly on the pretreatment before processing and before the mechanical testing. In particular, the tensile strength decreases with increasing the content of absorbed moisture in both the pretreatments. The elongation at break is strongly dependent on both the pretreatments, but, in this case, the elongation at break increases with increasing the amount of the absorbed moisture on both the pretreatments. In fact, the elongation at break is the mechanical property more dependent on the modifications of the molecular structure, on the modification of the morphology, and on the possible presence of water. The elongation at break increases, as expected, with increasing the amount of water in the specimen being the same as the pretreatment of the pellets before processing (from 65% for MC_11_ to 147% for MC_12_, to 301% for MC_13_), as well as with increasing the moisture absorbed before compression molding, being the same the pretreatment before testing (from 65% for MC_11_ to 82% for MC_21_, to 105% for MC_31_). The increase of the elongation at break is, however, larger when the moisture is absorbed before processing. The mechanical properties depend, therefore, on both the conditioning of the pellets before processing and on the conditioning of the specimens before mechanical testing. The effect of moisture content during the compression molding is to be attributed to the hydrolytic degradation with reduction of the molecular weight. The presence of moisture in the solid state gives rise to a plasticizing effect of the polymer.

**Figure 5:**
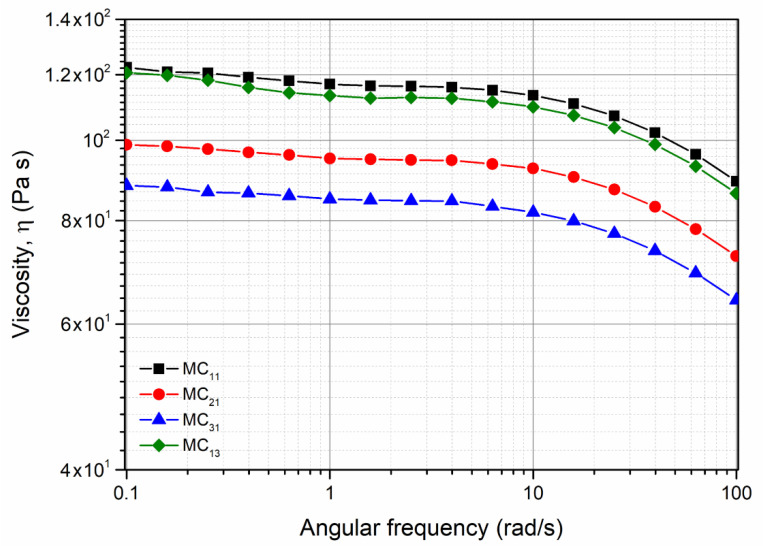
Flow curves of the samples from pellets conditioned under the three humidity conditions and specimen conditioned at RH = 0 and 90% after processing.

In Figure 5, the flow curves of the dry specimens MC_11_, MC_21,_ MC_31_, and of the sample MC_13_ are reported in order to verify a possible degradation of the polymer during processing as a consequence of the preliminary treatment of the pellets in different humidity conditions. The sample MC_13_ is reported for comparison and in order to verify a possible hydrolytic degradation when the moisture is absorbed after the processing. For the sake of simplicity, the flow curves of all the other specimens are not reported. The flow curves show that the viscosity decreases with increasing moisture content absorbed by the pellets before the processing. On the contrary, no significant effect on the viscosity is observed for the specimen conditioned after processing. This means that no significant effect of the conditioning before the measurement is observed on the viscosity.

As is well known, the Newtonian viscosity is strongly dependent on the molecular weight (see Equation (2)).

η_0_ = K Mw^3.4^ (2)

and, then, the reduction of the Newtonian viscosity means a reduction of the molecular weight, which can be calculated using Equation (3) [22] as follows:

(η_0_(MC_ij_)/η_0_ (MC_11_))^1/3.4^ = Mw(MC_11_)/Mw(MC_ij_) (3)

where *i* represents the condition of the pretreatment of the pellet and *j* represents the condition of the pretreatment of the specimens. This means that the decrease of the molecular weight for MC_21_ and MC_31_ is about 6.2% and 9.3%, respectively. The decrease of the molecular weight can be attributed to the hydrolysis undergone by the macromolecular chains due to the presence of water. As for the sample MC_13_, the flow curve is almost superimposable to that of the sample MC_11_, and this means that the humidity absorbed on the molded sample MC_13_ does not change the molecular structure of the polymer.

As already said, the increase of the elongation at break and the decrease of the rigidity observed for the humid specimens could be attributed to the presence of moisture in the specimens which acts as a plasticizer [23].

In order to verify better the effect of the pretreatment—before processing or after processing—the dimensionless values of the elongation at break were considered for all the samples. As previously mentioned, the elongation at break is the mechanical property more sensible to the change of molecular structure and morphology and to the presence of the water acting as a plasticizer.

**Figure 6:**
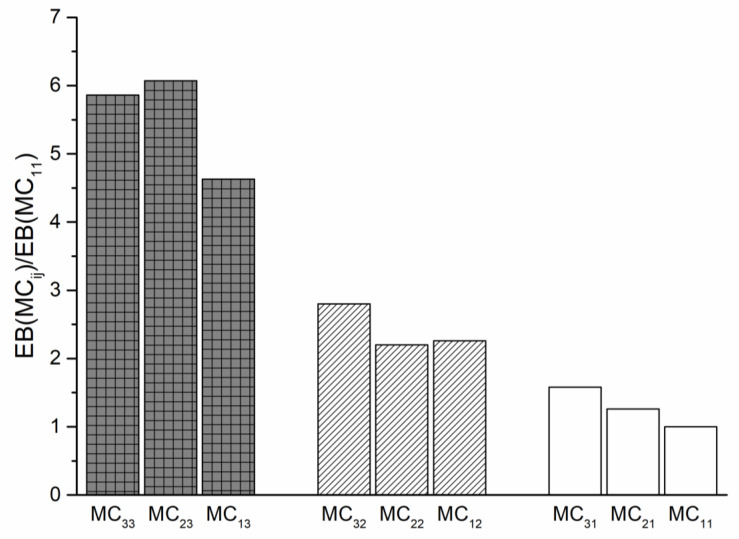
Histogram of the EB dimensionless values of all samples.

In Figure 6, the dimensionless values of EB are plotted for all the samples. The dimensionless values have been calculated as the elongation at break for each sample divided that of the sample MC_11_, i.e., the sample processed and characterized in a dry state.

It is possible to put in evidence three “blocks.” The largest increase of the elongation at break is observed for the samples treated in condition 3, i.e., samples exposed to the humidity after processing and before the testing. The lowest values of the dimensionless elongation at break are shown by the specimen conditioned in dry conditions. This, of course, means that in the investigated pretreatment conditions, the plasticizing effect of the water is more important than the decrease of the molecular weight as a consequence of the hydrolytic degradation.

## 4. Conclusions

The presence of moisture in the biodegradable polyesters both before processing and during their lifetime can strongly modify the rheological and mechanical properties of the polymers. In this work, the effect of moisture during the processing and the effect of different level of moisture in molded samples has been investigated. During processing, the presence of moisture decreases the molecular weight of the polymer and then the viscosity of the melt. As expected, the reduction of the molecular weight is higher with increasing the level of moisture because of the hydrolysis cleavage of the macromolecular chains. The presence of moisture in solid state slightly reduces the rigidity of the polymer and remarkably increases the deformability. This behavior has been attributed to the plasticizing effect of moisture. As for the effect of moisture on the mechanical behavior and, in particular, on the elongation at break, the effect of the presence of moisture in the samples seems to be predominant over the effect of the hydrolytic degradation during processing.

## Figures and Tables

**Table 1:** Environmental conditions used for conditioning the pellets and the compression-molded specimens.

Condition 1	Condition 2	Condition 3
T = 38 °C	T = 38 °C	T = 38 °C
RH = 0%	RH = 50%	RH = 90%

**Table 2:** Specimens for rheological and mechanical characterization.

Specimens Code		
MC_11_	MC_21_	MC_31_
MC_12_	MC_22_	MC_32_
MC_13_	MC_23_	MC_33_

**Table 3:** Moisture content of the polymer conditioned under different relative humidity.

Relative Humidity, %	Moisture Content, %
0	0.00
50	0.35
90	0.42

**Table 4:** Elastic modulus, E, tensile strength, TS, elongation at break, EB, of all the specimens.

Specimens Code	E, MPa	TS, MPa	EB, %
MC_11_	336 ± 18	13.0 ± 0.8	65 ± 6
MC_21_	334 ± 13	13.0 ± 0.7	82 ± 4
MC_31_	333 ± 13	12.0 ± 0.6	103 ± 10
MC_12_	329 ± 22	11.0 ± 0.8	147 ± 24
MC_22_	328 ± 17	11.5 ± 0.6	143 ± 15
MC_32_	323 ± 11	11.0 ± 0.8	182 ± 12
MC_13_	318 ± 10	10.5 ± 0.9	301± 29
MC_23_	314 ± 13	10.1 ± 1.1	395 ± 34
MC_33_	313 ± 18	9.0 ± 0.3	381 ± 16

## Data Availability

The data presented in this study are available on request from the corresponding author.
